# Compendiums of cancer transcriptomes for machine learning applications

**DOI:** 10.1038/s41597-019-0207-2

**Published:** 2019-10-08

**Authors:** Su Bin Lim, Swee Jin Tan, Wan-Teck Lim, Chwee Teck Lim

**Affiliations:** 1 NUS Graduate School for Integrative Sciences & Engineering, National University of Singapore, Singapore, Singapore; 2 Department of Biomedical Engineering, National University of Singapore, Singapore, Singapore; 3 Regional Scientific Affairs, Sysmex Asia Pacific, Singapore, Singapore; 4 Division of Medical Oncology, National Cancer Centre Singapore, Singapore, Singapore; 5 Office of Academic and Clinical Development, Duke-NUS Medical School, Singapore, Singapore; 6 IMCB NCC MPI Singapore Oncogenome Laboratory, Institute of Molecular and Cell Biology (IMCB), A*STAR, Singapore, Singapore; 7 Mechanobiology Institute, National University of Singapore, Singapore, Singapore; 8 Institute for Health Innovation and Technology (iHealthtech), National University of Singapore, Singapore, Singapore

**Keywords:** Data integration, Cancer genomics, Transcriptomics

## Abstract

There are massive transcriptome profiles in the form of microarray. The challenge is that they are processed using diverse platforms and preprocessing tools, requiring considerable time and informatics expertise for cross-dataset analyses. If there exists a single, integrated data source, data-reuse can be facilitated for discovery, analysis, and validation of biomarker-based clinical strategy. Here, we present merged microarray-acquired datasets (MMDs) across 11 major cancer types, curating 8,386 patient-derived tumor and tumor-free samples from 95 GEO datasets. Using machine learning algorithms, we show that diagnostic models trained from MMDs can be directly applied to RNA-seq-acquired TCGA data with high classification accuracy. Machine learning optimized MMD further aids to reveal immune landscape across various carcinomas critically needed in disease management and clinical interventions. This unified data source may serve as an excellent training or test set to apply, develop, and refine machine learning algorithms that can be tapped to better define genomic landscape of human cancers.

## Abstract (summary)

**Table:** 

Measurement(s)	transcriptome
Technology Type(s)	digital curation
Factor Type(s)	cancer type • health status
Sample Characteristic - Organism	Homo sapiens

Machine-accessible metadata file describing the reported data: 10.6084/m9.figshare.9901763

## Background & summary

The Cancer Genome Atlas (TCGA) increasingly serves as a ‘training’ reference to apply machine learning algorithms, having comprehensive, well-curated genomic data of over 11,000 tumors across 33 major cancer types. In recent years, this rich resource combined with machine learning has facilitated the development of cancer classifier^1^, markers predictive of drug sensitivity^2^, histopathology image-based prognostic predictor^3^, and novel indices associated with oncogenic dedifferentiation^4^. There also exist vast datasets deposited at the National Center for Biotechnology Information (NCBI) Gene Expression Omnibus (GEO) in the form of microarray. Applying machine learning to exploit them, however, is not straightforward; they are often generated using diverse platforms and normalization tools, and are annotated with non-standardized texts and definitions. All of these features add computational complexity to the existing high-dimensional data, necessitating multiple and intricate analytics tools for data integration and analysis.

**Fig. 1:**
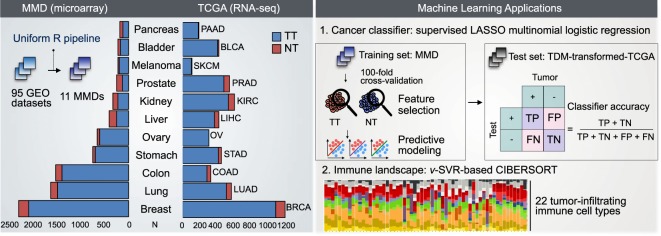
MMD: development, validation, and potential applications in oncology. Microarray-based datasets containing raw transcriptome profiles of patient-derived tumor tissues (TT) and non-tumor (NT) tissues were processed, merged, and batch-effect corrected using an integrated R pipeline. Validation of each cancer type-specific MMD was performed using PCA and RRHO algorithms. Clinical models trained using MMD can be applied to TCGA, facilitating the discovery of new biomarkers, development of prognostic models, and parallel cross-platform analyses with TCGA.

To increase the reuse of such legacy data, we generated single, merged microarray-acquired datasets (MMD) for 11 major cancer types using a uniform R pipeline (Fig. 1). This approach has been used in our earlier work to generate merged transcriptome data of a specific cancer type, non-small cell lung cancer (NSCLC), comprising both non-tumor (NT) and tumor tissue (TT) samples^5^. The resulting MMD was used to develop a predictive multi-gene classifier, termed as tumor matrisome index (TMi), for prognosis and prediction of response to adjuvant chemotherapy among NSCLC patients^6^.

Here, we extend the framework to include various carcinomas of epithelial origin. Consistent with prior works^7–11^, comparably correlated patterns of genome-wide differential expression (DE) were observed between microarray (MMD) and RNA-seq (TCGA). Next, we demonstrate the potential application of MMD as training data to develop clinical predictive models that can be applied cross platform. By applying CIBERSORT^12^, we further show how MMDs can be used to de-convolve tumor immune microenvironment by parsing specific subpopulations of infiltrating immune cell, comparatively with TCGA datasets of matching cancer types.

Through pan-cancer analysis of MMDs, we recently identified clinically significant matrisomal changes associated with immune response and targetable immune checkpoints for a subset of cancers across different malignancies^13^. The generated cancer type-specific MMDs, the associated clinical metadata and R codes are available at ArrayExpress and figshare (see Data Records and Code Availability). Our open resource of curated large-scale transcriptomic data may provide the basis for the analytical and computational techniques to derive unbiased and new information, enabling predictive modeling for precision oncology.

## Methods

### MMD generation

A careful GEO search (http://www.ncbi.nlm.nih.gov/geo) was done to ensure the selection of MIAME compliant datasets having the following attributes in the original GEO submission: (1) raw data in CEL files, (2) tissue origin annotation (i.e., NT or TT), and (3) Affymetrix platform annotation. Here, only datasets generated using the GPL570 platform (Affymetrix Human Genome U133 Plus 2.0 Array) were specifically selected to ensure uniform curation of the same probe-sets (i.e., 54,675 probes). Altogether, 95 independent GEO datasets comprising a total of 8,386 samples spanning over 11 cancer types were subjected to pre-processing, normalization, batch-effect correction, data integration and analyses (Table S1). The number of NT and TT samples in each GEO dataset is summarized in Table S2.

Raw expression data from each dataset was first imported and loaded into R Bioconductor^14^ (RStudio version 1.1.447) using the affy package (version 1.48.0)^15^. The ReadAffy function was called with default parameters to read all CEL files, except for the function argument “cdfname” which was set to “hgu133plus2”. The rma function was subsequently used to normalize and background correct all the annotated probe-sets-derived expression data. This preprocessing step was applied to all 95 datasets for uniform processing and feature annotation prior to merging based on cancer type. Batch effects were identified and removed using ComBat via the inSilicoMerging package (version 1.14.0)^16^. Probes having maximum mean expression values across samples in each MMD were collapsed to the genes, and were annotated using the hgu133plus2SYMBOL object in the hgu133plus2.db package (version 3.2.2)^17^ for subsequent DE analysis.

### TCGA datasets

The Cancer Genome Atlas (TCGA) data were retrieved and processed via the TCGA-Assembler package (version 2.0)^18^ (Table S1). Normalized RPKM count values were extracted using the ProcessRNASeqData function via the TCGA-Assembler package (version 2.0)^18^. Only genes with at least 1 count per million (cpm) or RPMK value in at least 20% of total number of samples in each cohort were kept via the edgeR package (version 3.12.1)^19^. The number of genes filtered out in each TCGA dataset is summarized in Table S3. Selected genes were normalized by Trimmed Mean of M-values (TMM), and were subjected to DE analyses using the voom and lmFit functions in the limma package (version 3.26.9)^20^. Of note, ovarian (OV) and melanoma (SKCM) TCGA cohorts were excluded in DE and RRHO analyses due to lack of NT samples (Table S1). Clinical data including disease status (NT vs. TT) were downloaded via the DownloadBiospecimenClinicalData function in the TCGA-Assembler package (version 2.0)^18^.

### PCA, DE and RRHO analysis

Principal component analysis (PCA) was performed using the prcomp function in the built-in R stats package (version 3.2.2). The first two PCs were visualized using the ggbiplot package (version 0.55)^21^. The lmFit and eBayes functions in the limma package (version 3.26.9)^20^ were used to perform DE analysis. All genes annotated in each MMD and TCGA dataset were ranked by log fold change (logFC) computed based on their DE between NT and TT samples. These ranked lists were further reconstructed to only include genes that were common to both MMD- and TCGA-derived lists^22^ (Table S3). These files were loaded into a web-based executable simplified version of rank-rank hypergeometric overlap (RRHO) tool (http://systems.crump.ucla.edu/rankrank/rankranksimple.php). In all cases, the step size was set to 300 to generate Benjamin-Yekutieli corrected hypergeometric matrix and RRHO heatmaps.

### Multi-gene classifiers

Expression data of TMi and other gene signatures of commercially available or previously validated multi-gene tests (MGTs) were extracted from all TT samples across MMD and TCGA datasets, and were loaded into Morpheus (http://software.broadinstitute.org/morpheus/) for sample stratification. The list of MGT genes and the associated references are summarized in Table S4. K-means clustering was performed with “one minus pearson correlation” metric and 1,000 iterations.

### CIBERSORT

Consisting of over 1,500 samples, breast, colon, and lung MMDs exceeded the load capacity (500MB) of the CIBERSORT analysis (http://cibersort.standford.edu/)^12^. 1,000 samples were thus randomly selected to generate the input “mixture” file for these MMDs. All samples in the rest of MMDs were included in the CIBERSORT analysis. Each run was performed with a default LM22 (22 immune cell types) gene signature using 100 permutations. The resulting immune cell profiles were used to compute the mean fractions of 22 immune cell types and the quantitative change between the two groups (NT vs. TT), denoted as delta (TT – NT, %), per dataset.

### ROC analysis

A summary of four MGTs applied to MMDs, including gene signatures, the associated references, computation method for respective prognostic index, is provided in Table S5. Diagnostic accuracy of MGTs in classifying TT from NT samples was evaluated through the receiver operating characteristic (ROC) analysis. The area under the ROC curve (AUC), sensitivity, and specificity with the optimal cutoff for respective prognostic index were computed using the pROC package (version 1.10.0)^23^.

## Data Records

Our 11 MMDs are available at ArrayExpress for lung^24^, pancreas^25^, prostate^26^, kidney^27^, stomach^28^, colon^29^, ovary^30^, breast^31^, liver^32^, bladder^33^, and melanoma cancer^34^.

## Technical Validation

### Principal component analysis (PCA)

**Fig. 2:**
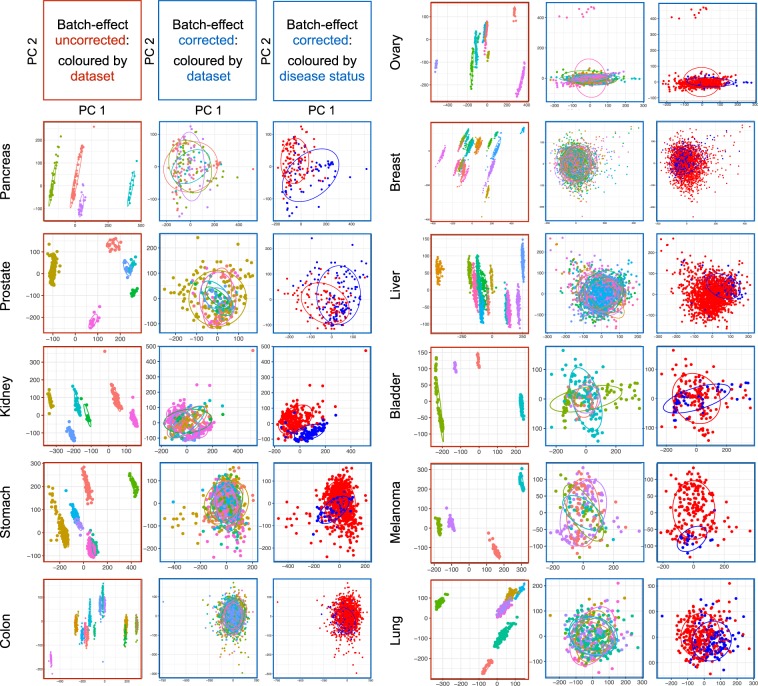
QC metrics for MMDs. The first two PCs capturing the most variance are shown. PCA plots with red colored-border show PCs of merged data before batch-effect correction, which are colored by dataset (left). PCA plots with blue colored-border show PCs of merged data after Combat adjustment, which are colored by dataset (middle) and disease status (i.e., TT vs. NT; right). Ellipses are drawn one standard deviation away from the mean of the Gaussian fitted to each MMD.

PCA was performed to assess the performance of ComBat in correcting batch effects, as previously described^6,35^. The first two PCs that capture the most variance are shown for both untransformed and ComBat-transformed datasets (Fig. 2). Batch-effect corrected MMDs exhibit an apparent overlay of PCs colored by the study (i.e., original dataset), and are separated by the disease status (i.e., NT vs. TT), demonstrating successful adjustment of batch effects arising from independent datasets of different sources. The PCA plots of MMD data exclusively comprising TT samples further distinguished the two risk groups (TMi_high_ and TMi_low_) stratified by a pan-cancer multi-gene TMi classifier (Fig. S1; see Methods).

### Differential expression (DE) analysis

Prior to in-depth genome-wide DE analysis, expression levels of cancer-related genes and three reference genes (i.e., GAPDH, UBB, and ACTB) were compared between the two groups (NT vs. TT) using MMDs. The selected housekeeping genes are stably expressed across tissues to maintain cellular function, and are commonly used for normalization in transcriptomics studies. While expression levels of cancer-associated gene were significantly different between NT and TT samples, that of all reference genes were almost the same in the two groups across all cancer types, validating the robustness of ComBat in adjusting technical batch effects while maintaining biological variation across samples (Fig. S2).

All MMDs were next subjected to genome-wide, limma-based DE analysis to rank all the genes by logFC based on DE between NT and TT samples (see Methods). These ranked lists were used to generate volcano plots visually depicting differentially expressed genes that met our statistical threshold (i.e., absolute value of logFC > 1 and adjusted P-value < 0.001) in TT relative to NT samples (Fig. S3 and Table S5). To validate these results in an independent cohort of patients, we processed TCGA data of matching cancer types (see Methods), and applied the same methods to construct the list of differentially expressed genes.

### Rank-rank hypergeometric overlap (RRHO) analysis

**Fig. 3:**
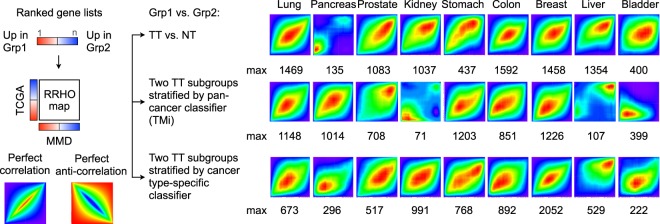
Parallel genome-wide differential expression (DE) analyses with TCGA. Rank-rank hypergeometric overlap (RRHO) heatmaps are drawn to visualize the overlap intensity between MMD- and TCGA-derived lists of genes ranked by DE between the two groups: TT vs. NT group (top row), between the two TT subgroups classified by TMi (middle row) and by known cancer type-specific classifier (bottom row). RRHO map max values, denoted as max, are stated.

RRHO algorithm^36^ was used to assess the overlap intensity between MMD- and TCGA-derived lists of genes ranked by DE between NT and TT samples per cancer type (Fig. 3). As compared to conventional single arbitrary cut-off-based approaches, RRHO heatmaps have been widely used to visually compare genome-wide DE patterns across different species and profiling platforms, without having to correct for batch effects for the two distinct data files^36,37^. A significant overlap was observed for lung, prostate, kidney, colon, breast, and liver cancer, for which RRHO map max ranged from 1083 for kidney cancer to 1592 for colorectal cancer (Fig. 3, top row). The weak correlation observed across pancreas, stomach, and bladder cancers between MMD and TCGA datasets is likely due to a relatively small number of tumor-free tissues available in respective TCGA datasets (Table S1).

To test whether this would indeed be the case, we utilized the TMi annotation (TMi_high_ or TMi_low_) previously derived from MMD data exclusively comprising TT samples (Fig. S1), and further classified TMi group for all TCGA TT samples using the same approaches (Table S3; see Methods). Except for bladder cancer, RRHO map max increased significantly from 135 to 1014 for pancreatic cancer and 437 to 1203 for gastric cancer (Fig. 3, middle row). Similarly, highly concordant RRHO results were derived from TT subgroups stratified by other commercially available or previously validated cancer type-specific multi-gene classifiers (Fig. 3, bottom row; see Methods). These QC steps altogether demonstrate the robustness of our uniform workflow for cross-cancer analysis (Fig. S4).

### Machine learning applications for predictive medicine

#### Cancer classifier

Publicly-accessible data repositories, such as GTEx^38^, TCGA^39^, HPA^40^, and ArrayExpress^41^, host genome-wide expression profiles assayed with various profiling technologies. Having sufficient read depth^10^, higher resolution^11^, higher dynamic range^42^, and lower technical variation^43^, RNA-seq is increasingly the platform of choice in translational-biomarker studies. Paralleling this trend, cross-platform normalization tools continue to be developed, facilitating comparison of data from different platforms. PREBS^44^, VOOM^45^, and TDM^42^ are examplary techniques that are specifically designed to transform RNA-seq data to make it compatible with microarray data. Other conventional methods also exist in dealing with such ‘dataset shifts’^46^, such as quantile normalization, log_2_ transformation, and nonparanormal transformation^42^.

**Fig. 4:**
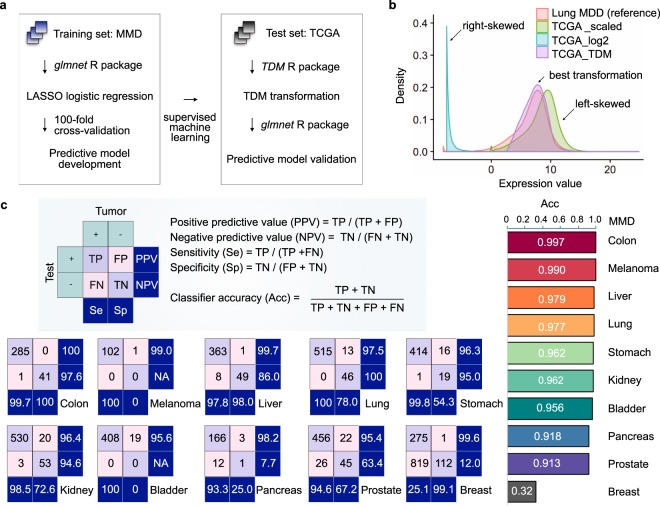
Supervised machine learning classifies cancer. (**a**) Schematic workflow: cancer classifiers are built from MMDs, and are tested on TCGA of matching cancer types using LASSO logistic regression. (**b**) TDM-transformed testing data (TCGA LUAD) best fits the training data distribution (lung MMD). (**c**) Classifying accuracy of MMD-derived cancer classifier.

Using supervised machine learning, we developed new cancer classifiers trained on MMDs, and evaluated their classifying performance on their respective RNA-seq-acquired TCGA datasets (Fig. 4a). Among the existing transformation methods, TDM transformation best fitted the reference MMD data distribution (Fig. 4b). Using the glmnet package (version 2.0.13)^47^, we performed LASSO multinomial logistic regression^48^ with 100 fold cross-validation (CV) to build best predictive model in distinguishing TT from NT samples. Predictive model built from each MMD was then tested directly on TDM-transformed-TCGA dataset. Except for breast MMD, all MMDs achieved an average AUC of 0.96 (ranging from 0.913 to 0.997) in classifying TCGA cancers (Fig. 4c). Other commercially available MGTs, including the Myriad myplan^TM^ Lung Cancer, Pervenio^TM^, Oncotype DX and MammaPrint, further achieved the AUC ranging from 0.714 to 0.862 (Table S6, Fig. S5; see Methods).

#### Pan-cancer immunogenomic analyses

TCGA data are increasingly being used to study the prognostic influence of the composition of tumor-infiltrating lymphocytes (TILs)^49,50^, neoantigens^51,52^ and immune cytolytic activity^53^, all of which are putative markers predictive of clinical response to immune checkpoint inhibitor (ICI) treatments. The recent advancements in computational techniques have further facilitated high-resolution, large-scale immunogenomic analyses of the tumor-immune interface^54^. Of the developed analytical pipelines, CIBERSORT serves as an exemplary *in silico* deconvolution method to estimate the relative proportion of 22 immune cell populations from heterogeneous bulk tissues. By applying CIBERSORT to MMDs, we next tested if the generated compendiums could further provide the basis for the developed computational infrastructure to reveal clinically significant immune landscape across multiple cancer types (see Methods).

**Fig. 5:**
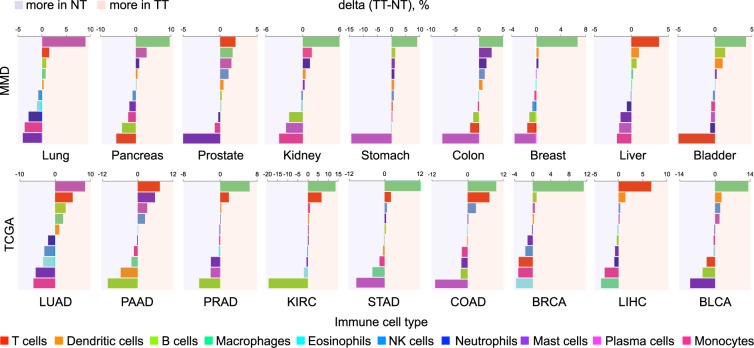
Immune cell composition in NT and TT samples. Quantified changes of CIBERSORT-estimated fractions of immune cell populations between the two groups using MMD (top) and TCGA (bottom) datasets.

The extent of difference in immune cell composition between the two groups (NT vs. TT) varied depending on cancer type (Fig. S6), where the estimated fractions were generally comparable (<5% difference). Specific immune cell types particularly enriched in either NT or TT group were identified, including plasma cells in lung cancer, T cells in liver cancer, and B cells in kidney, stomach, colon, breast, and bladder cancers (Fig. 5). Their enrichment was further observed in respective TCGA datasets, demonstrating the potential use of MMDs to reveal the degree and distribution of TIL density, which might be a clinically relevant prognostic and predictive indicator across various carcinomas^55,56^.

## Supplementary Information

Supplementary Information

## Data Availability

The R codes used to preprocess, merge, and correct for batch-effects for generation of all 11 cancer type-specific MMDs can be found in figshare (10.6084/m9.figshare.7878086)^22^. The exemplary R codes and metadata used to develop clinical predictive models using lung MMD^57^ are described in our earlier works^5,6,58^.
