# Supplementary material for: Compendiums of cancer transcriptomes for machine learning applications
Source: Sci Data. 2019 Oct 8;6:194. doi: 10.1038/s41597-019-0207-2 (PMC6783425; doi:10.1038/s41597-019-0207-2)
Supplement: Supplementary file 1 — Supplementary Information [file 41597_2019_207_MOESM1_ESM.pdf]

Supplementary Figure

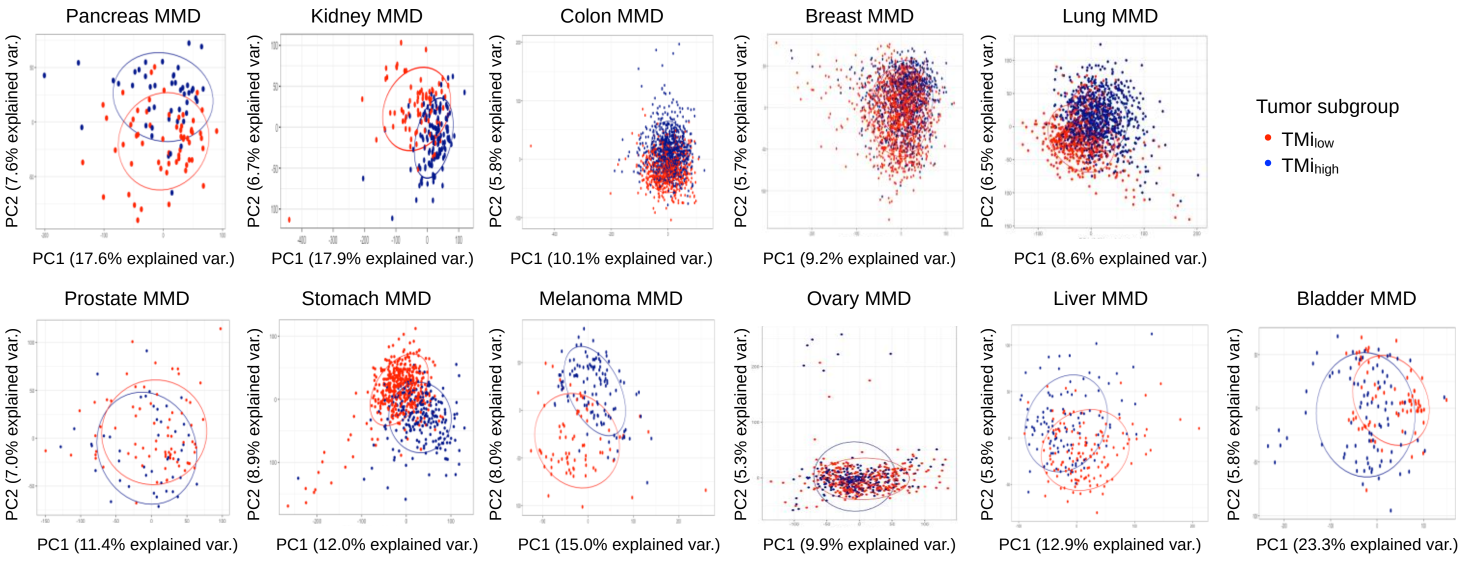

Fig. S1. PCA of TT subgroups. All TT samples in MMDs were stratified into TMi_{low} and TMi_{high} groups based on 29-gene matrisome expression (see Methods). The first two PCs capturing the most variance are shown. Ellipses are drawn one standard deviation away from the mean of the Gaussian fitted to each MMD.

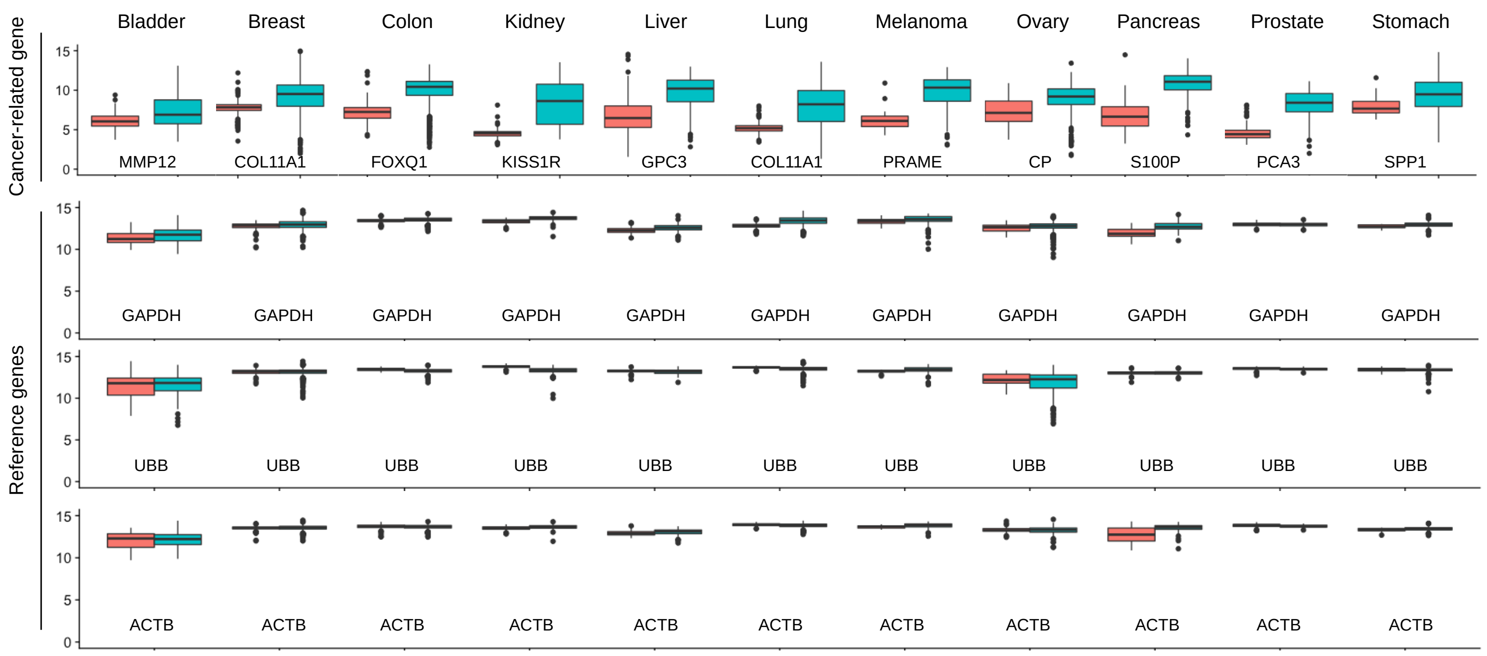

Fig. S2. Box plots comparing the expression of cancer-related genes and reference genes between NT and TT samples. Box hinges represent 1st and 3rd quartiles, and middle represents the median. The upper and lower whiskers extend from hinges up and down indicate the most extreme values that are within 1.5*IQR (interquartile range) of the respective hinge. Red and blue boxes represent non-tumor (NT) and tumor tissue (TT) samples, respectively.

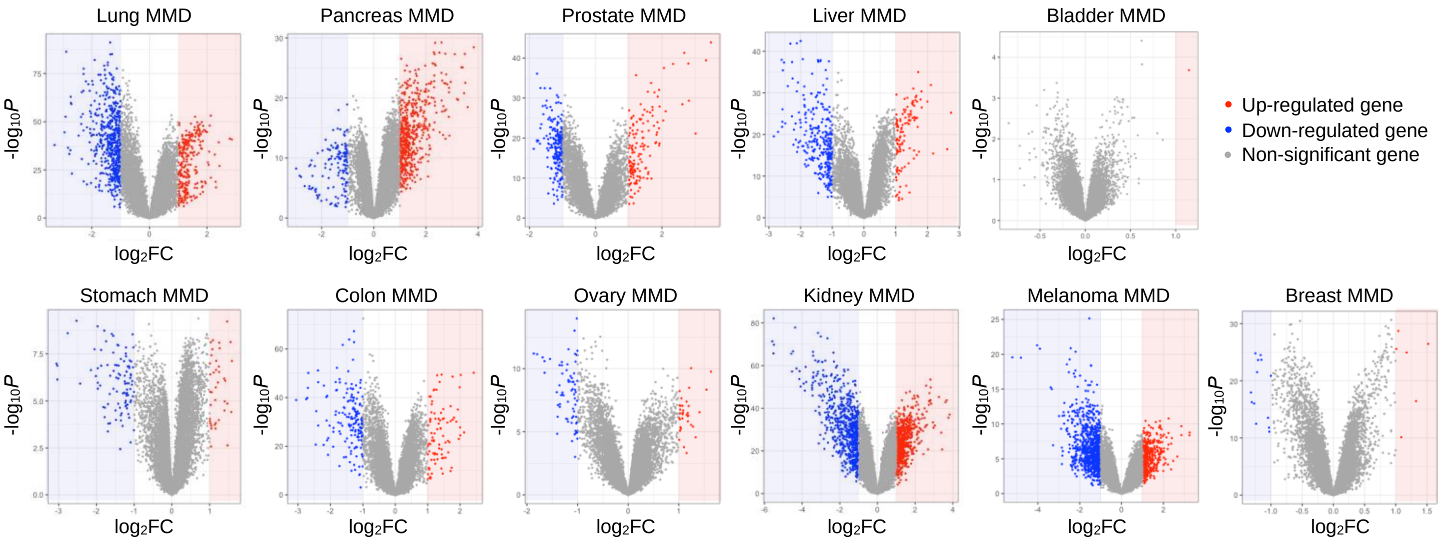

Fig. S3. Differential expression analysis. Up-regulated and down-regulated genes with absolute values of log2 fold-change>1 and LIMMA-derived adjusted P-value<0.001 in TT samples relative to NT samples are shown in red and blue, respectively. Gray dots represent statistically non-significant genes.

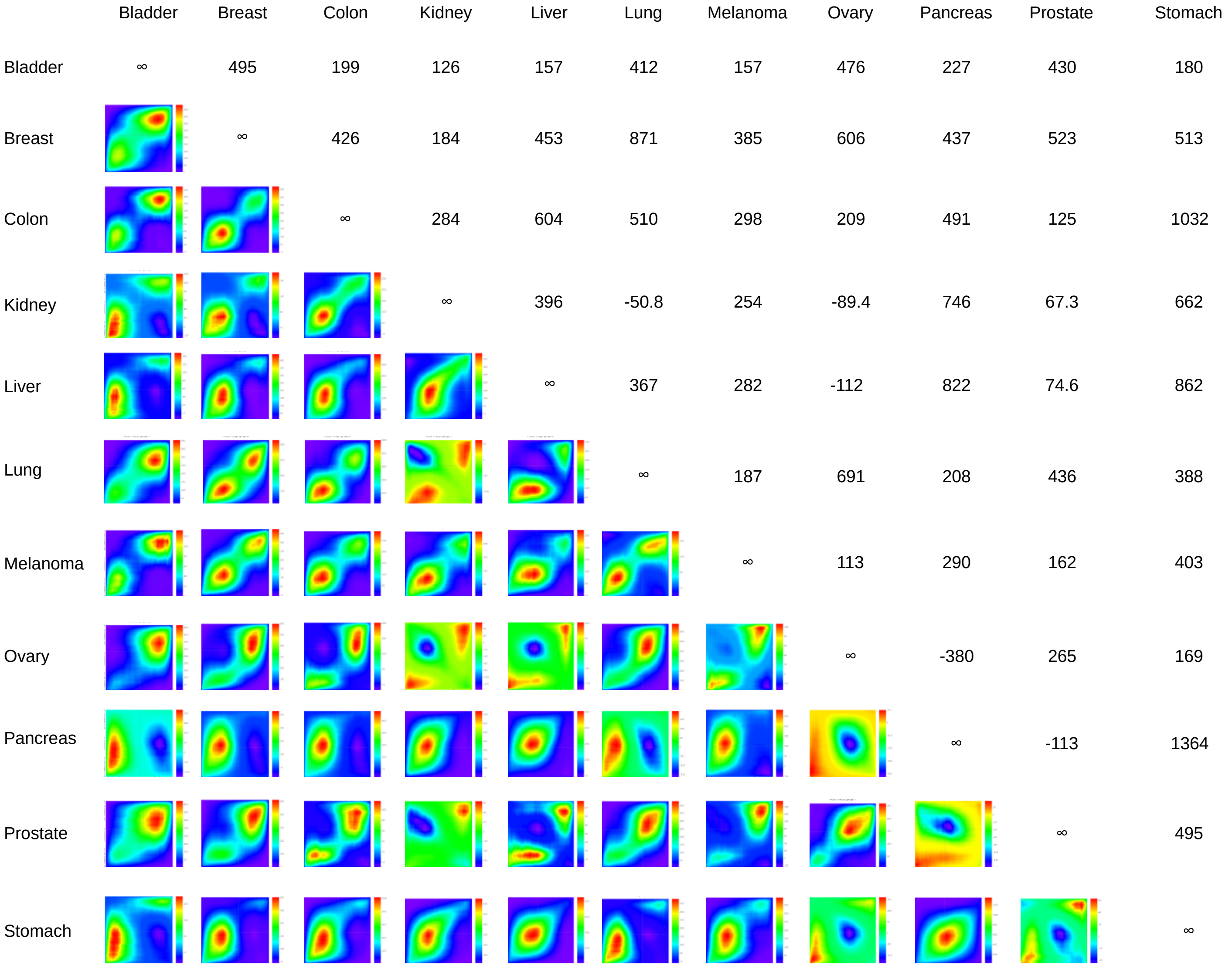

Fig. S4. Cross-cancer analysis. Lists of genes ranked by DE between TT and NT groups were used.to generate RRHO heatmaps. RRHO map max values, denoted as max, are stated.

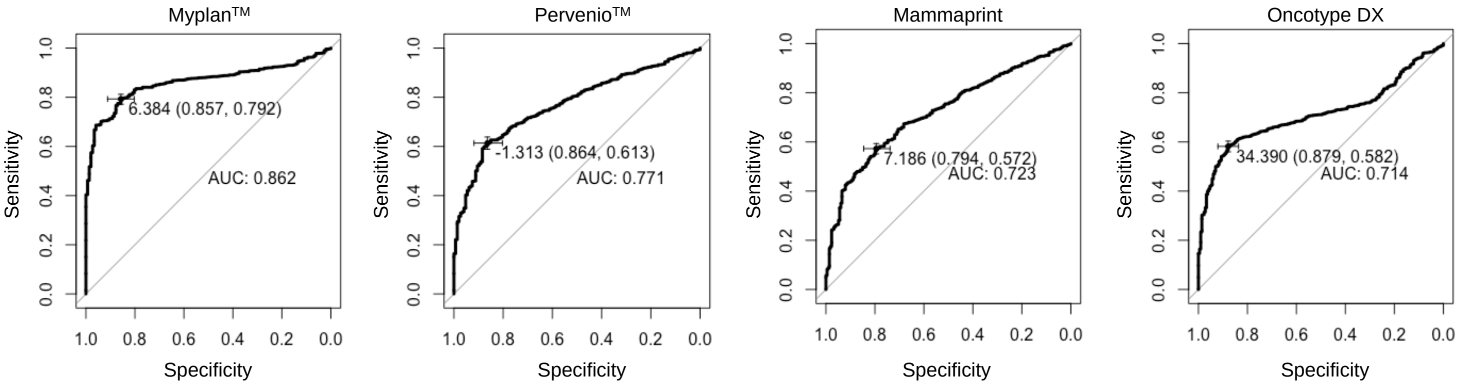

Fig. S5. ROC analysis. The optimal cutoff, specificity, sensitivity, and the area under the ROC curve (AUC) are stated for each multi-gene classifier.

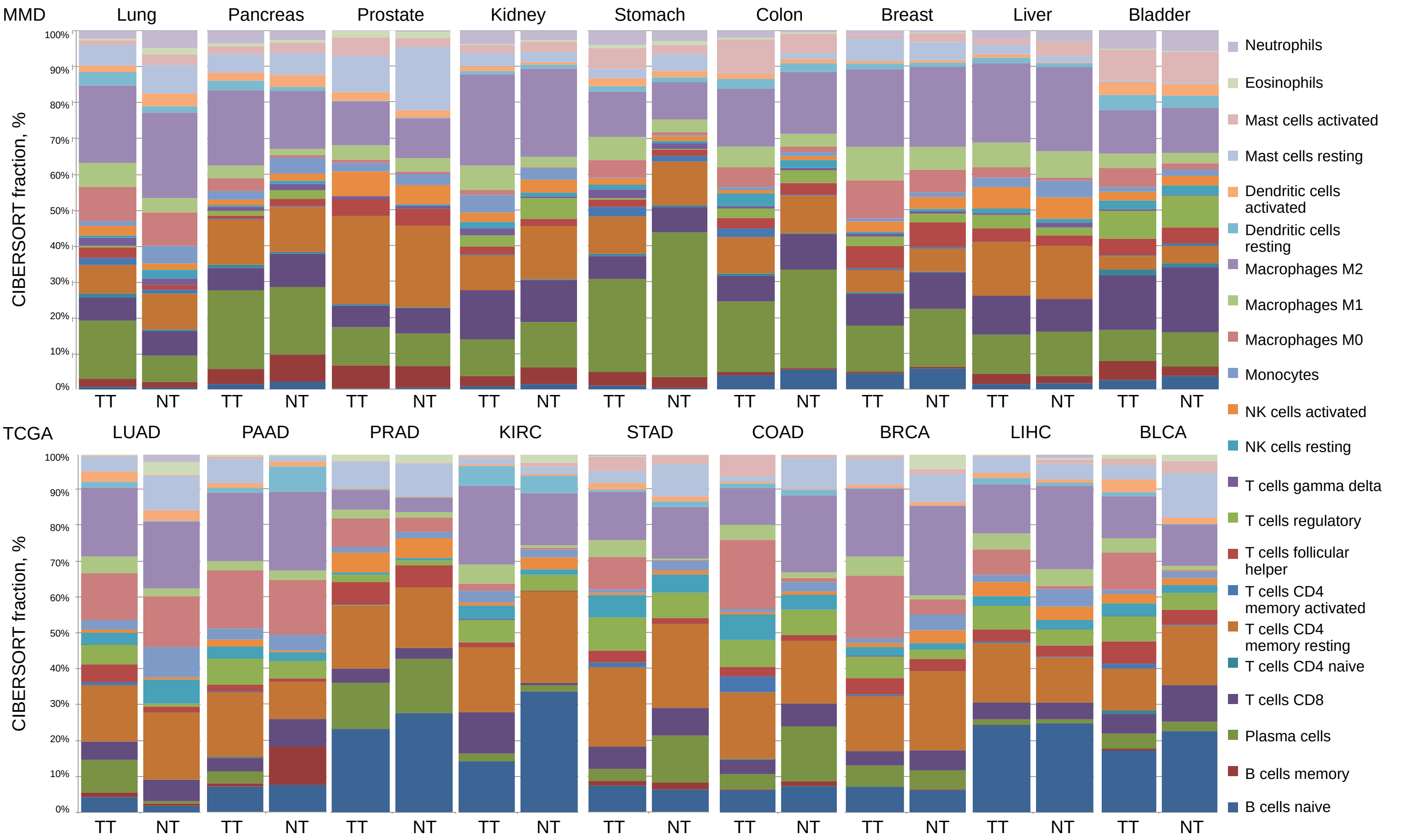

Fig. S6. CIBERSORT analysis. The relative fractions of 22 immune cell types are shown for TT and NT group across MMD (top) and TCGA (bottom) datasets.

Supplementary Tables

Table S1. Summary of MMD and TCGA datasets. N = sample size; TT = tumor tissue; NT = non-tumor tissue.

Cancer type	Lung	Pancreas	Prostate	Kidney
GEO accession codes	GSE10245	GSE15471	GSE17906	GSE36895
	GSE10445	GSE16515	GSE17951	GSE23629
	GSE10799	GSE22780	GSE26910	GSE11151
	GSE12667	GSE32676	GSE3325	GSE53757
	GSE18842		GSE45016	GSE12606
	GSE19188		GSE55945	GSE12090
	GSE28571			
	GSE30219			
	GSE31210			
	GSE33356			
	GSE37745			
	GSE50081			
Total N	1621	178	237	323
TT N	1474	108	121	219
NT N	147	70	116	104

TCGA	LUAD	LUSC	PAAD	PRAD	KICH	KIRC	KIRP
Total N	574	553	182	549	90	606	323
TT N	515	502	178	482	65	533	291
NT N	59	51	4	67	25	73	32

Cancer type	Stomach	Colon	Ovary	Breast	Liver	Bladder	Melanoma
GEO accession codes	GSE13911	GSE13067	GSE10971	GSE10780	GSE29721	GSE30522	GSE15605
	GSE15459	GSE13294	GSE12172	GSE12276	GSE40873	GSE31189	GSE19234
	GSE19826	GSE14333	GSE14001	GSE16446	GSE41804	GSE31684	GSE23376
	GSE34942	GSE15960	GSE14407	GSE18864	GSE45436	GSE7476	GSE7553
	GSE35809	GSE17536	GSE15578	GSE19615	GSE6222		
	GSE38749	GSE17537	GSE18520	GSE20685	GSE62232		
	GSE62254	GSE18088	GSE19352	GSE20711	GSE75271		
		GSE18105	GSE19829	GSE21653			
		GSE20916	GSE30161	GSE22513			
		GSE23878	GSE9899	GSE23177			
		GSE26682		GSE23593			
		GSE33113		GSE26639			
		GSE4107		GSE28796			
		GSE4183		GSE28821			
		GSE9348		GSE3744			
				GSE42568			
				GSE50567			
				GSE5764			

				GSE7904<br>GSE9195			
Total N	737	1514	647	2302	401	212	214
TT N	691	1393	593	2088	244	161	194
NT N	46	121	54	214	157	51	20
TCGA	STAD	COAD	OV	BRCA	LIHC	BLCA	SKCM
Total N	450	327	304	1207	421	427	103
TT N	415	286	304	1094	371	408	102
NT N	35	41	0	113	50	19	1

Table S2. Summary of 95 GEO datasets used in MMD generation. N = sample size; TT = tumor tissue; NT = non-tumor tissue.

Organ	Dataset	NT N	TT N	Organ	Dataset	NT N	TT N	Organ	Dataset	NT N	TT N
Lung	GSE10245	0	58	Colon	GSE9348	12	70	Bladder	GSE7476	3	9
Lung	GSE10445	0	72	Pancreas	GSE15471	39	39	Breast	GSE10780	143	42
Lung	GSE10799	3	16	Pancreas	GSE16515	16	36	Breast	GSE12276	0	204
Lung	GSE12667	0	75	Pancreas	GSE22780	8	8	Breast	GSE16446	0	120
Lung	GSE18842	45	46	Pancreas	GSE32676	5	25	Breast	GSE18864	0	84
Lung	GSE19188	65	91	Prostate	GSE17906	10	12	Breast	GSE19615	0	115
Lung	GSE28571	0	100	Prostate	GSE17951	86	68	Breast	GSE20685	0	327
Lung	GSE30219	14	293	Prostate	GSE26910	0	6	Breast	GSE20711	2	88
Lung	GSE31210	20	226	Prostate	GSE3325	0	13	Breast	GSE21653	0	266
Lung	GSE33356	0	120	Prostate	GSE45016	1	10	Breast	GSE22513	0	28
Lung	GSE37745	0	196	Prostate	GSE55945	7	0	Breast	GSE23177	0	116
Lung	GSE50081	0	181	Stomach	GSE13911	31	38	Breast	GSE23593	0	50
Kidney	GSE11151	5	62	Stomach	GSE15459	0	200	Breast	GSE26639	0	226
Kidney	GSE12090	0	18	Stomach	GSE19826	15	12	Breast	GSE28796	0	24
Kidney	GSE12606	4	6	Stomach	GSE34942	0	56	Breast	GSE28821	0	89
Kidney	GSE23629	0	32	Stomach	GSE35809	0	70	Breast	GSE3744	7	40
Kidney	GSE36895	23	29	Stomach	GSE38749	0	15	Breast	GSE42568	17	104
Kidney	GSE53757	72	72	Stomach	GSE62254	0	300	Breast	GSE50567	6	35
Colon	GSE13067	0	74	Liver	GSE29721	10	10	Breast	GSE5764	20	10
Colon	GSE13294	0	155	Liver	GSE40873	49	0	Breast	GSE7904	19	43
Colon	GSE14333	0	290	Liver	GSE41804	20	20	Breast	GSE9195	0	77
Colon	GSE15960	0	6	Liver	GSE45436	41	93	Ovary	GSE10971	24	13
Colon	GSE17536	0	177	Liver	GSE6222	2	10	Ovary	GSE12172	0	90
Colon	GSE17537	0	55	Liver	GSE62232	10	81	Ovary	GSE14001	0	20
Colon	GSE18088	0	53	Liver	GSE75271	5	50	Ovary	GSE14407	12	12
Colon	GSE18105	27	94	Melanoma	GSE15605	16	46	Ovary	GSE15578	6	4
Colon	GSE20916	44	91	Melanoma	GSE19234	0	44	Ovary	GSE18520	10	53
Colon	GSE23878	24	35	Melanoma	GSE23376	0	22	Ovary	GSE19352	2	20
Colon	GSE26682	0	176	Melanoma	GSE7553	4	82	Ovary	GSE19829	0	28
Colon	GSE33113	6	90	Bladder	GSE30522	8	7	Ovary	GSE30161	0	58
Colon	GSE4107	10	12	Bladder	GSE31189	40	52	Ovary	GSE9899	0	295
Colon	GSE4183	8	15	Bladder	GSE31684	0	93				

Table S3. Summary of MMD and TCGA data processing and overlapping genes. N = sample size.

TCGA dataset	Initial gene N	After filtering	MMD dataset	Initial gene N	Overlapping gene N with TCGA ranked list of differentially expressed genes
LUAD	20474	15404	Lung	20546	14101
PAAD	20474	15533	Pancreas	20546	14239
PRAD	20474	14976	Prostate	20546	13720
KIRC	20474	15131	Kidney	20545	13852
STAD	20474	15418	Stomach	20546	14104
COAD	20474	14785	Colon	20546	13571
BRCA	20474	15165	Breast	20546	13887
LIHC	20474	14138	Liver	20546	13022
BLCA	20474	15033	Bladder	20546	13771
OV	20474	15480	Ovary	20546	NA
SKCM	20474	14747	Melanoma	20546	NA

Table S4. Summary of multi-gene classifiers used in DE and RRHO analyses.

Original signature	Kidney MMD	TCGA KIRC	Reference
NR3C2	NR3C2	NR3C2	Bhalla S, Chaudhary K, Kumar R, Sehgal M, Kaur H, Sharma S, Raghava GP: Gene expression-based biomarkers for discriminating early and late stage of clear cell renal cancer. Sci Rep 2017, 7:44997.
ENAM	ENAM	ENAM	
DNASE1L3	DNASE1L3	DNASE1L3	
MAP6D1	MAP6D1	MAP6D1	
SMPD4		SMPD4	
C11orf73			
PLEKHA9			
FRMPD2			

Original signature	Stomach MMD	TCGA STAD
NR1I2	NR1I2	NR1I2
LGALSL	LGALSL	LGALSL
C1ORF198	CST2	CST2
CST2	LAMP5	LAMP5
LAMP5	FOXS1	FOXS1
FOXS1	CES1P1	MMP7
CES1P1	MMP7	COL8A1
MMP7	COL8A1	
COL8A1		

Original signature	Lung MMD	TCGA LUAD	Reference
BAG1	BAG1	BAG1	Kratz JR, He J, Van Den Eeden SK, Zhu ZH, Gao W, Pham PT, Mulvihill MS, Ziaei F, Zhang H, Su B, et al.: A practical molecular assay to predict survival in resected non-squamous, non-small-cell lung cancer: development and international validation studies. Lancet 2012, 379:823-832.
BRCA1	BRCA1	ERBB3	
CDC6	CDC6	FUT3	
CDK2AP1	CDK2AP1	WNT3A	
ERBB3	ERBB3	BRCA1	
FUT3	FUT3	CDC6	
IL11	IL11	CDK2AP1	
LCK	LCK	IL11	
RND3	RND3	LCK	
SH3BGR		RND3	
WNT3A			

Original signature	Bladder MMD	TCGA BLCA
S100A8	S100A8	S100A8
CELSR3	CELSR3	CELSR3
PFKFB4	PFKFB4	PFKFB4
HMOX1	HMOX1	HMOX1
MTAP	MTAP	MTAP
MGC17624	KIF1A	KIF1A
KIF1A	COCH	COCH
COCH		

Original signature	Pancreas MMD	TCGA PAAD	Reference
GPR87	GPR87	GPR87	Birnbaum DJ, Finetti P, Lopresti A, Gilabert M, Poizat F, Raoul JL, Delpero JR, Moutardier V, Birnbaum D, Mamessier E, et al.: A 25-gene classifier predicts overall survival in resectable pancreatic cancer. BMC Med 2017, 15:170.
KRT13	KRT13	KRT13	
RAC2	RAC2	RAC2	
C16orf74	C16orf74	C16orf74	
NAMPT	NAMPT	NAMPT	
DHRS9	DHRS9	DHRS9	
HIST2H2BF	TREM2	HIST2H2BF	
TREM2	ZDHHC20	TREM2	
ZDHHC20	CD180	ZDHHC20	
CD180	ADGRG6	CD180	
ADGRG6	APBB1IP	ADGRG6	
APBB1IP	EGR3	APBB1IP	
EGR3	MACROD2	EGR3	
MACROD2	EPHA7	MACROD2	
EPHA7	RASGEF1A	EPHA7	
RASGEF1A	SYNM	RASGEF1A	
SYNM	S100A1	SYNM	
S100A1	WNK2	S100A1	
WNK2	RAMP2	WNK2	
RAMP2	SOCS2	RAMP2	

Original signature	Colon MMD	TCGA COAD
MCTP1	MCTP1	MCTP1
LAMA3	LAMA3	LAMA3
CTSC	CTSC	CTSC
PYROXD1	PYROXD1	PYROXD1
EDEM1	EDEM1	EDEM1
IL2RB	IL2RB	IL2RB
ZNF697	ZNF697	ZNF697
SLC6A11	SLC6A11	IL2RA
IL2RA	IL2RA	CYFIP2
CYFIP2	CYFIP2	PIM3
PIM3	PIM3	LIF
LIF	LIF	PLIN3
PLIN3	PLIN3	ZBED4
HSD3B1	HSD3B1	PPARA
ZBED4	ZBED4	THNSL2
PPARA	PPARA	
THNSL2	THNSL2	
CA4388O2		

Original signature	Liver MMD	TCGA LIHC
AHCYL2	AHCYL2	AHCYL2

SOCS2	COL28A1	SOCS2		LAMP2	LAMP2	LAMP2
COL28A1	B4GALT6	COL28A1		SPRY1	SPRY1	SPRY1
B4GALT6	PLCB4	B4GALT6		SERPINA7	SERPINA7	SERPINA7
PLCB4	MTURN	PLCB4		FGGY	FGGY	FGGY
MTURN		MTURN		ASLNC16648		
**Original signature**	**Prostate MMD**	**TCGA PRAD**	**Reference**	**Original signature**	**Breast MMD**	**TCGA BRCA**
LASP1	LASP1	LASP1	Erho N, Crisan A, Vergara IA, Mitra AP, Ghadessi M, Buerki C, Bergstralh EJ, Kollmeyer T, Fink S, Haddad Z, et al.: Discovery and validation of a prostate cancer genomic classifier that predicts early metastasis following radical prostatectomy. PLoS One 2013, 8:e66855.	AURKA	LASP1	MMP11
IQGAP3	IQGAP3	IQGAP3		BAG1	IQGAP3	GRB7
NFIB	NFIB	NFIB		BCL2	S1PR4	ERBB2
S1PR4	S1PR4	S1PR4		BIRC5	THBS2	ESR1
THBS2	THBS2	THBS2		CCNB1	EPPK1	PGR
ANO7	ANO7	ANO7		CD68	NUSAP1	BCL2
PCDH7	PCDH7	PCDH7		CTSL2	ZWILCH	SCUBE2
MYBPC1	MYBPC1	MYBPC1		ERBB2	UBE2C	GSTM1
EPPK1	EPPK1	EPPK1		ESR1	NFIB	BAG1
TSBP	PBX1	PBX1		GRB7	ANO7	MKI67
PBX1	NUSAP1	NUSAP1		GSTM1	PCDH7	AURKA
NUSAP1	ZWILCH	ZWILCH		MKI67	MYBPC1	BIRC5
ZWILCH	UBE2C	UBE2C		MMP11	PBX1	CCNB1
UBE2C	CAMK2N1	CAMK2N1		MYBL2	CAMK2N1	MYBL2
CAMK2N1	RABGAP1	RABGAP1		PGR	RABGAP1	CD68
RABGAP1	TNFRSF19	TNFRSF19		SCUBE2	TNFRSF19	
PCAT-32						
GLYATL1P4/PCAT-80						
TNFRSF19						

Table S5. Summary of MMD-derived differentially expressed genes. N = sample size.

Cancer type	Up-regulated genes N	Down-regulated gene N	Non-significant genes N
Bladder	1	0	20545
Breast	6	13	20527
Colon	86	167	20293
Stomach	39	99	20408
Liver	113	289	201
Melanoma	392	860	19294
Ovary	35	68	20443
Pancreas	570	136	19840
Prostate	147	223	20176
Lung	267	555	19724
Kidney	662	743	19141

Table S6. Summary of multi-gene classifiers used in ROC analyses.

Cancer type	MGT	Original signature	Lung or Breast MMD	Reference	Computation method
Lung	myplan™	ASPM, CDCA8, MCM10, FOXM1, CDC20, CDKN3, BIRC5, DLGAP5, KIF20A, BUB1B, PRC1, TK1, CEP55, PBK, RAD54L, NUSAP1, RRM2, KIAA0101, ORC6L, RAD51, CENPM, SKA1, CENPF, KIF11, PTTG1, CDC2, DTL, PLK1, CDCA3, ASF1B, TOP2A	ASPM, CDCA8, MCM10, CDC20, CDKN3, BIRC5, DLGAP5, KIF20A, BUB1B, PRC1, TK1, CEP55, PBK, RAD54L, NUSAP1, RRM2, KIAA0101, RAD51, CENPM, SKA1, CENPF, KIF11, PTTG1, DTL, PLK1, CDCA3, ASF1B, TOP2A	Bueno, R. et al. Validation of a molecular and pathological model for five-year mortality risk in patients with early stage lung adenocarcinoma. J Thorac Oncol 10, 67-73, (2015).	Unweighted average
Lung	Pervenio	BAG1, BRCA1, CDC6, CDK2AP1, ERBB3, FUT3, IL11, LCK, RND3, SH3BGR, WNT3A	BAG1, BRCA1, CDC6, CDK2AP1, ERBB3, FUT3, IL11, LCK, RND3, SH3BGR	Kratz, J. R. et al. A practical molecular assay to predict survival in resected non-squamous, non-small-cell lung cancer: development and international validation studies. Lancet 379, 823-832, (2012).	Expression value of each gene was multiplied by predefined Cox proportional hazards model coefficients and the sum of these values represents the final index
Breast	MammaPrint	AA555029_RC, AKAP2, ALDH4A1, AP2B1, AYTL2, BBC3, C16orf61, C20orf46, c9orf30, CCNE2, CDC42BPA, CDCA7, CEGP1, CENPA, COL4A2, DCK, DIAPH3, DTL, EBF4, ECT2, EGLN1, ESM1, EXT1, FGF18, FLT1, GMPS, GNAZ, GPR126, GPR180, GSTM3, HRASLS, IGFBP5, JHDM1D, KNTC2, LGP2, LIN9, LOC100131053, LOC100288906, MCM6, MELK, MMP9, MS4A7, MTDH, NMU, NUSAP1, ORC6L, OXCT1, PAML2, PECI, PITRM1, PRC1, QSCN6L1, RAB6B, RASSF7, RECQL5, RFC4, RTN4RL1,	AA555029_RC, AP2B1, CCNE2, CDC42BPA, CDCA7, CENPA, COL4A2, DCK, DIAPH3, DTL, EBF4, ECT2, EGLN1, ESM1, EXT1, FGF18, FLT1, GMPS, GNAZ, GPR180, GSTM3, HRASLS, IGFBP5, LIN9, LOC100131053, MCM6, MELK, MMP9, MS4A7, MTDH, NMU, NUSAP1, OXCT1, PAML2, PITRM1, PRC1, RAB6B, RASSF7, RECQL5, RFC4, RTN4RL1, RUNDC1, SLC2A3, STK32B, TGFB3, TSPYL5, UCHL5, WISP1	Marquart, J., Chen, E. Y. & Prasad, V. Estimation of the Percentage of US Patients With Cancer Who Benefit From Genome-Driven Oncology. JAMA Oncol 4, 1093-1098, (2018).	Unweighted average

		RUNDC1, SERF1A, SLC2A3, STK32B, TGFB3, TSPYL5, UCHL5, WISP1, ZNF533			
Breast	Oncotype DX	AURKA, BAG1, BCL2, BIRC5, CCNB1, CD68, CTSL2, ERBB2, ESR1, GRB7, GSTM1, MKI67, MMP11, MYBL2, PGR, SCUBE2	AURKA, BAG1, BCL2, BIRC5, CCNB1, ERBB2, ESR1, GRB7, GSTM1, MKI67, MMP11, MYBL2, PGR, SCUBE2	Sparano, J. A. et al. Prospective Validation of a 21-Gene Expression Assay in Breast Cancer. The New England journal of medicine 373, 2005-2014, (2015).	Expression value of each gene was multiplied by predefined Cox proportional hazards model coefficients and the sum of these values represents the final index
